# Patient understanding of two commonly used patient reported outcome measures for primary care: a cognitive interview study

**DOI:** 10.1186/s12875-018-0850-2

**Published:** 2018-09-27

**Authors:** Mairead Murphy, Sandra Hollinghurst, Chris Salisbury

**Affiliations:** Centre for Academic Primary Care, Bristol Medical School, University of Bristol, Canynge Hall, 39 Whatley Road, Bristol, BS8 2PS UK

**Keywords:** Questionnaires, Primary care, Cognitive interviews, Patient-reported outcomes

## Abstract

**Background:**

Standardised generic patient-reported outcome measures (PROMs) which measure health status are often unresponsive to change in primary care. Alternative formats, which have been used to increase responsiveness, include individualised PROMs (in which respondents specify the outcomes of interest in their own words) and transitional PROMs (in which respondents directly rate change over a period). The objective of this study was to test qualitatively, through cognitive interviews, two PROMs, one using each respective format.

**Methods:**

The individualised PROM selected was the Measure Yourself Medical Outcomes Profile (MYMOP). The transitional PROM was the Patient Enablement Instrument (PEI). Twenty patients who had recently attended the GP were interviewed while completing the questionnaires. Interview data was analysed using a modification of Tourangeau’s model of cognitive processing: comprehension, response, recall and face validity.

**Results:**

Patients found the PEI simple to complete, but for some it lacked face validity. The transitional scale was sometimes confused with a status scale and was problematic in situations when the relevant GP appointment was part of a longer episode of care. Some patients reported a high enablement score despite verbally reporting low enablement but high regard for their GP, which suggested hypothesis-guessing. The interpretation of the PEI items was inconsistent between patients.

MYMOP was more difficult for patients to complete, but had greater face validity than the PEI. The scale used was open to response-shift: some patients suggested they would recalibrate their definition of the scale endpoints as their illness and expectations changed.

**Conclusions:**

The study provides information for both users of PEI/MYMOP and developers of individualised and transitional questionnaires.

Users should heed the recommendation that MYMOP should be interview-administered, and this is likely to apply to other individualised scales. The PEI is open to hypothesis-guessing and may lack face-validity for a longer episode of care (e.g. in patients with chronic conditions). Developers should be cognisant that transitional scales can be inconsistently completed: some patients forget during completion that they are measuring change from baseline. Although generic questionnaires require the content to be more general than do disease-specific questionnaires, developers should avoid questions which allow broad and varied interpretations.

**Electronic supplementary material:**

The online version of this article (10.1186/s12875-018-0850-2) contains supplementary material, which is available to authorized users.

## Background

Assessing the effectiveness of healthcare interventions from a patient perspective involves the use of patient-reported outcome measures (PROMs). Many PROMs are disease-specific, that is, tailored to the symptoms and impacts on function of a particular condition [1]. However, primary care services are first contact, comprehensive and co-ordinating [2] which means that patients can have a range of conditions. Many primary care studies thus require a *generic* PROM: one which can be administered regardless of condition [3]. A key problem with many generic PROMs is that they are limited to symptoms and function. Primary care patients frequently present with other problems, [4] and many have long-term conditions [5] whereby improvement in function may be unlikely. Leading generic PROMs such as the SF-36 [6] and EQ-5D [7], therefore often show no change following interventions in primary care [8–10].

In this context, the authors of this paper set out to develop a new PROM for primary care which would be more responsive to change than other PROMs. A 24-item PROM was developed, the Primary Care Outcomes Questionnaire (PCOQ). The qualitative development work [11–13] and psychometric testing [14] of this have been published elsewhere. This article reports on a study which was carried out as part of the qualitative development work. Some PROMs have been designed to increase responsiveness when used in primary care [10, 15, 16] and the researchers decided to carry out primary research to assess two of these, before embarking on development of the PCOQ. Patients who were interviewed for the qualitative study were asked to complete these two existing questionnaires designed to measure outcomes in primary care and the process of completion was assessed using cognitive interviews.

Cognitive interviews are based on the theory that responding to a questionnaire involves a number of cognitive tasks [17]. The most common theory to explain this process originates with Tourangeau [18] and was further developed by Willis [19]. It describes the cognitive tasks which people go through when responding to questionnaires as consisting of four components: comprehension, retrieval, decision and response. Questionnaire respondents must firstly *comprehend* the question and secondly *retrieve* information from memory, or form a judgement on the spot. They then *decide* what they wish to share with the researcher before fitting their own mental *response* to the categories provided by the questionnaire [17].

The purpose of cognitive interviews is not just to understand these cognitive processes, but to use this to detect potential sources of response error. Through use of such a model, cognitive interviews can successfully identify problems with questionnaires which can then be corrected before the data are collected from a larger sample. Despite the fact that this is an essential element of all PROM development, many PROM developers miss this important stage out, or provide only a cursory report on it [20].

Two formats, which are often more responsive than standardised generic formats, are firstly PROMs with transitional scales and secondly individualised PROMs. Transitional scales capture outcome without the need for a baseline, relying on the patient remembering their health status before the intervention and directly assessing their level of change. For example, a common generic transitional item is “thinking about the main problem you consulted your doctor with, is this problem…”, with response options given on a five-point Likert scale from very much better to very much worse [21].

Individualised PROMs allow patients to specify their problems themselves. They are therefore focussed on the issues particular to the patient in question and thus show change when other PROMs do not [10].

The objective of this study was to test qualitatively, through cognitive interviews, two PROMs designed specifically for primary care: a PROM which uses a transitional scale and an individualised PROM.

## Methods

### Research setting

The research setting was the National Health Service (NHS) in the UK. As described above, this study was carried out alongside a larger qualitative study, which had been designed to explore patients’ and practitioners’ views on the most important outcomes arising from primary care consultations [11]. The larger qualitative study was itself carried out to inform development of a PROM, which has since been quantitively tested [22]. The current study received ethical approval from Nottingham 1 National Research Ethics Service (NRES) [23]. Patients were recruited from the waiting rooms of three health centres in areas of varying deprivation and one walk-in centre in Bristol. Patients were purposefully sampled to include both men and women, and a range of ages, conditions and ethnicities. Immediately following their semi-structured interview for the main qualitative study, patients who were willing to do so also completed a cognitive interview to test the two PROMs.

### Selection of instruments

The selected PROMs were the patient enablement instrument (PEI) and the measure yourself medical outcome profile version 2 (MYMOP). These were selected because they were both designed to address the limitations of generic PROMs in relation to measuring outcome in primary care.

#### PEI instrument overview

**Fig. 1:**
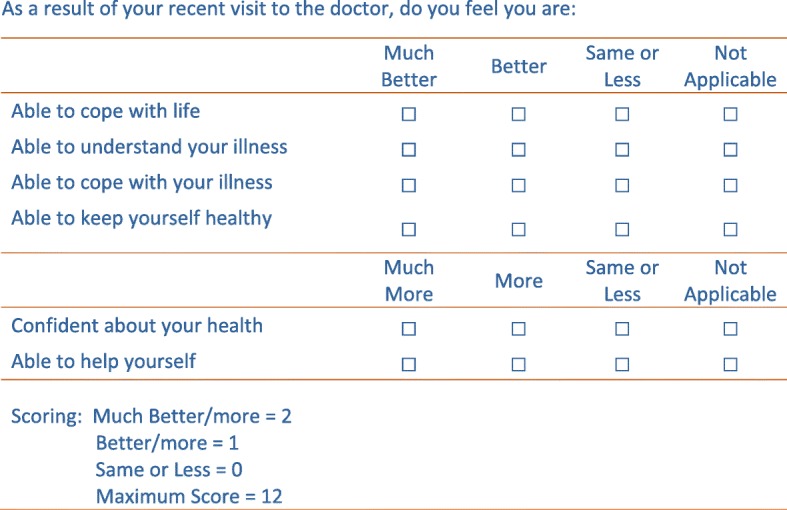
Patient enablement instrument

The PEI was the first PROM to be developed specifically for primary-care and is based on the principle that the key purpose of primary care consultations is to ‘enable’ patients to better live their lives and understand and manage any conditions they may have. It was originally designed as a broader instrument, to measure the input, processes and outcomes of high-quality primary care. The PEI retained six items from the original instrument, which capture the construct “enablement”, comprising elements of empowerment, understanding and coping [15]. It is a transitional instrument which aims to measure increase in enablement resulting from a single consultation. The scoring precludes decreased enablement. The PEI is shown in Fig. 1.

The PEI has undergone extensive psychometric testing and has been widely translated and used in different countries [24]. It has shown good test-retest reliability [25] and internal consistency [15]. Construct validity has been demonstrated through correlation of the PEI with patient empowerment in patients with multiple long-term conditions [26]. The PEI also correlates with  measures of patient experience, such as the doctor’s communication skills, [27] knowing the doctor well, [15, 24] consultation length [24, 28] and receiving a prescription when desired [24, 29].

The PEI is normally completed straight after a GP consultation. A modified version of the PEI was used in this study which used the words “as a result of your recent visit to the doctor/nurse.”

#### MYMOP instrument overview

MYMOP was the first individualised instrument developed to measure outcomes in primary care. Development was influenced by the Patient-Generated Index, [30] driven by the fact that primary care patients have different conditions, symptoms and priorities, so outcomes measurement in primary care needs to be similarly individualised. MYMOP measures symptoms, activity and well-being at a point in time. The symptoms and activity section is individualised and related to a single (as opposed to multiple) health problem; respondents identify the problem-related symptoms and activity which are most important to them. Individualised tools in general have weaker psychometric properties than do standardised [31–33]. At its initial development, cogent criticisms were made of the derivation and mathematical principles underlying MYMOP [34, 35]. Since then, MYMOP has been widely used and further validated, in particular with patients accessing alternative and complementary therapies, such as acupuncture or homeopathy [36–38].

Although it has been used as a self-completed instrument in a limited number of trials, [39, 40] the developer of the MYMOP has recommended that it is completed during a consultation with a patient through a structured interview [41]. A follow-up questionnaire is completed at an agreed time in the future (also by interview) and the score calculated as the change from baseline status.

A modified version of MYMOP was used in this study, whereby the patient name and address was removed from the top of the questionnaire and questions on medication were removed from the bottom. These changes are specified in the MYMOP website as allowable without compromising validity [41].

**Fig. 2:**
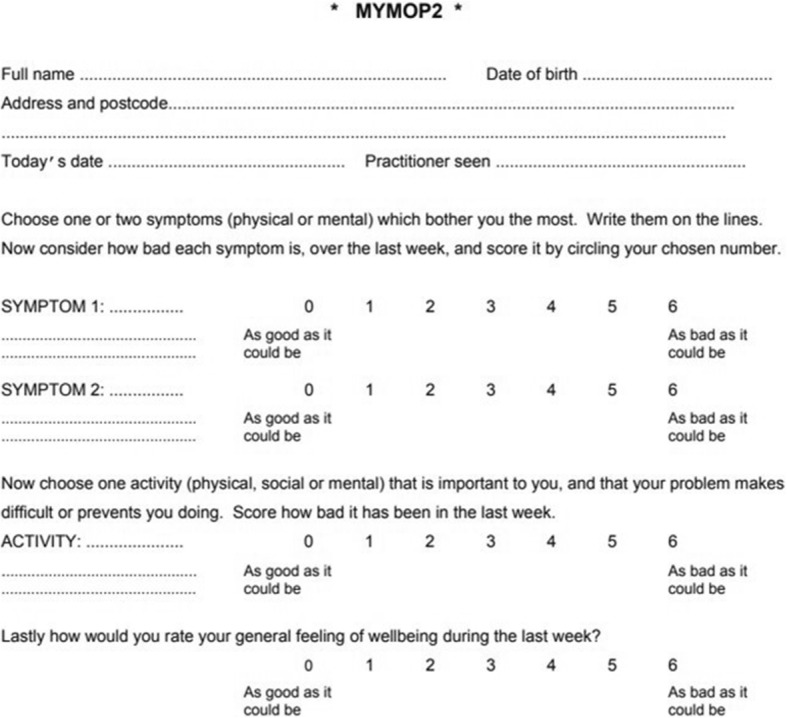
MYMOP

The section of MYMOP given to respondents is shown Fig. 2.

### Data collection

Data were collected through cognitive interviews: a method whereby people are interviewed as they complete questionnaires and asked to explain the cognitive processes used when arriving at an answer. Cognitive interviewing is often used to improve questionnaire design [19]. In this study, it was used to understand the benefits and limitations of these two PROMs, in particular the respective use of individualised items and a transitional scale. There are two main methods for conducting a cognitive interview: verbal probing (in which the interviewer probes respondents as they complete a questionnaire) and think-aloud (in which respondents describe their cognitive processes as they complete a questionnaire without intervention from the interviewer) [42].

To minimise burden on patients, [19] this study primarily used the verbal probing method. If patients began to naturally think-aloud, this was adopted in addition to verbal probes. The topic guide is shown in Additional file 1.

### Data analysis

The cognitive interviews were audio-recorded and transcribed verbatim. One researcher [MM] read and re-read the interview transcripts in order to gain an overall view of the accounts given, to identify themes and develop an initial coding framework based on the Torangeau model. Tourangeau’s model [18] was adjusted because, in common with most other studies, we found few problems with the decision process [43, 44]. We therefore focussed on the other three processes, as follows:

1. **Comprehension Process:** Does the respondent understand what is intended by the question?
2. **Retrieval Process:** Is the respondent able to retrieve the information from memory correctly from the correct time-period?
3. **Response process:** Does the respondent manage to map their desired response onto the scale without introduction of error? For example, do they understand the scale and are the scale responses available appropriate?

To finalise the coding frame, the two co-investigators [CS&SH] independently reviewed four interview transcripts and identified themes within the above three categories. CS, SH and MM then discussed these themes and agreed on the coding frame. MM then electronically coded all the interviews according to this coding frame using a spreadsheet-based tabular format. If a problem was identified, this was mapped to the coding framework using memos and the verbatim quotes to justify the decision. We also assessed face validity: whether the questionnaire appeared to respondents “on the face of it” to capture what it is purported to capture, and whether their responses gave a fair reflection of their situation.

COREQ guidelines were followed in carrying out the data collection and analysis [45].

## Results

### Sample and respondents

Twenty people completed a cognitive interview for the PEI. Three of these did not complete MYMOP because they thought it was not relevant to them as they had not had any symptoms when they attended for a consultation. The characteristics of the 20 participants is shown in Table 1. Comprehension, recall and response process problems are shown in Table 2.

**Table 1:** Patient characteristics

Characteristic	Number
Gender	
Female	13
Male	7
Age Bracket	
18–34	5
35–54	5
55–64	3
65–74	4
75+	3
Ethnicity	
Asian	1
Black	1
Mixed race	1
White	17
Number LTCs	
No long-term conditions	4
One long-term condition	7
> One long-term condition	9

**Table 2:** PEI and MYMOP Comprehension, Response and Recall problems

PROM	Cognitive Process	Problem Area	Total patients^a^
PEI (*n* = 20)	Compre-hension	Patient expressed uncertainly on how to interpret item	5
		Patient left an applicable item blank or ticked n/a	1
		*Total patients with at least one problem*	*6*
	Recall	Scored overall improvement since start of illness, not because of doctor.	6
		Difficulty keeping the score to a single appointment, within an episode of care	6
		*Total patients with at least one problem*	*9*
	Response	Suggested same or less category should be split into two	5
		Difficulty choosing between “same or less” / “not applicable”	4
		Data entry error made as a result of response scale	5
		*Total patients with at least one problem*	*9*
MYMOP (*n* = 17)	Compre-hension	Difficulty with choosing, or sticking to a single “problem”	4
		Symptoms misunderstood as conditions	8
		Activity misunderstood as sporting / paid work	3
		*Total people with at least one problem*	*8*
	Recall	Difficulty averaging over a week for something that is cyclical or has changed over the week.	3
		*Total people with at least one problem*	*3*
	Response	Patient expressed confusion on how to interpret scale	4
		Patient interpreted scale inconsistently between questions	2
		*Total people with at least one problem*	*6*

^a^some patients reported more than one type of problem in each process

PEI items = 1) Able to cope with life, 2) Able to understand your Illness, 3) Able to cope with your illness, 4) Able to keep yourself healthy, 5) Confident about your health, 6) Able to help yourself

MYMOP items = 1) Two symptoms (individualised) 2) One activity (individualised) 3) Overall well-being

### Patient enablement instrument

#### Comprehension

The PEI interviews were characterised by very different interpretations for each of the six questions. There is no documentation available on how the PEI items should be interpreted, so these different readings were not counted as “problems” in Table 2, as it is not clear which interpretation is correct. However, such ambiguity clearly does demonstrate a problem with the questionnaire; and the different interpretations are listed by item in Table 3. Four people commented that they found the questions “vague” or “open”. Others hesitated before responding and highlighted possible different interpretations. Dual interpretations were found for most questions, generally with one broad interpretation and one more narrowly focussed on the consultation. For example, 13 patients interpreted an impaired ability to “cope with life” as being depressed, or having lost the motivation or ability to function. One participant explained: “If you’re not [able to cope with life] you’ve had it, haven’t you?” Five considered any alleviation of minor concern or symptoms as improving their ability to cope with life. One participant became frustrated deciding how to interpret this saying: “*it’s a real ‘nothing’ sentence. Isn’t it?”(Patient 9)* Similarly, the question “able to help yourself” was interpreted by some as able to take any action to improve symptoms or problems, and others as an absence of helplessness: i.e. ability to function.

**Table 3:** PEI dual interpretation of items

PEI item	Interpretation of item	Total
Able to cope with life	Not “coping” means depressed or unable to go about day to day tasks	13
	Improved “coping” can be any reduction in minor concern.	5
Able to understand illness	My long-term condition is not an “illness”	3
	My short-term condition is not an “illness”	5
	I do / did have an illness when I consulted the doctor / nurse	12
Able to cope with your illness	Taking practical action to make the illness less problematic	9
	Includes reduction of concern even if no practical action taken	7
Able to keep yourself healthy	Refers to general diet, exercise, well-being	7
	Refers to the particular problem consulted for	8
Confident about your health	Increased understanding / confidence in managing condition	7
	Confidence in diagnosis and management plan	3
	Confident that condition can be dealt with / is not serious	7
	Confident that you an overall healthy person	2
Able to help yourself	Able to manage in daily life (not helpless)	8
	Any actions taken to improve or alleviate symptoms / condition	11

The use of the word “illness” was the subject of some discussion. Eight people felt they were not “ill”. This included both people with long-term conditions (epilepsy, polycystic ovaries, heart condition with valve fitted), and those with short term conditions (allergic rash, bruised leg, baker’s cyst) as well as a woman who was pregnant.

*“I don’t really think of my heart as an illness funny enough. It's not like suddenly it can be cured. I suppose you think your illness is something which you'll hopefully get over it. Whereas the heart, guess I'm stuck with that.” (Patient 5)*

Most people who raised this issue were, however, still comfortable with responding to the item as if their problem were an illness.

#### Recall process

The recall process is how patients retrieve the necessary information from memory [19]. Some patients found the transitional nature of the scale difficult. These mostly consisted of people with on-going issues which were not fully resolved by their last doctor’s appointment. Six respondents found it difficult to identify whether improvements resulted from primary care or their own self-management. Patient 16 dealt with this by giving two sets of responses: one for how much she had improved because of her own actions (unrelated to the consultation) and one for how much she had improved because of the consultation.

Six patients also had difficulty rating a single consultation during an episode of care, rather than the improvement delivered through all primary care consultations since the start of the episode. One respondent caveated the answer she had given as follows:

*Patient 18: “Yeah. I suppose these things are all sort of, like rather than specifically today, or yesterday … it’s as a whole I suppose.”*

*INT: “Yeah. Do you find it difficult to separate out yesterday because it’s a recurrent appointment?”*

*Patient 18: “Yeah. [INT: Yeah.] Yeah because …. I always see the same lady and it’s always about the same thing.”*

Other respondents similarly referred to their improvement since the start of an episode, or repeatedly sought confirmation from the interviewer that the item referred to improvements only related to the last appointment.

#### Response process

Nine people had problems with mapping their response to the scale. Five respondents made errors with the “same or less” category. One respondent scored out the word “less” on the questionnaire. Three respondents ticked “better” when the correct response was “same” but they did not want to create confusion that their response might be “less”:

*INT: “As a result of your visit to the doctor two weeks ago do you feel you are able to cope with life ‘much better’, ‘better’, ‘same or less’, or ‘not applicable’?”*

*Patient 10: “I have a comment here. [INT: Mm.] Wouldn’t it have been better to have ‘much better’, ‘better’, ‘same’, then ‘less’?”*

*INT: “Mm, some people have said that.”*

*Patient 10: “Yes. Because it is … they’re not the same thing. And … because of that I’m going to put ‘better’ rather than ‘the same’ […] it WAS the same. You see. So I can’t … but I can’t say ‘the same’ […] because it … it might be interpreted as less. And that would make it wrong.”*

Four people commented that they had similar difficulties in choosing between “not applicable” and “same or less” and one respondent, following the heuristic of a Likert scale, completed the questionnaire believing “not applicable” read “much less.”

#### Face validity

Face validity is the extent to which a questionnaire appears to be measuring what it is in fact measuring [46]. A number of people questioned the relevance of the PEI. One respondent, who said she thought the questionnaire looked like a “waste of time”, justified this by the ambiguity of the questions:

*I might have ticked different boxes actually if I’d have done it straight away after my appointment. […] In that situation, I probably would have just (mimes self ticking without thought) and because they’re so almost vague and open […] I just wouldn’t have sat and focused on it. (Patient 4)*

Another respondent, who said “*for me that is a useless questionnaire”,* explained that she felt unable to give meaningful responses because of the grouping of “Same or Less” into a single response option.

Face validity is not always desirable [31]. In some cases, the PEI seemed to possess too much face validity, leading to hypothesis-guessing, with patients seeing PEI as an assessment of the GP and responding based on this assessment rather than a change in enablement. For example, one woman, who held her GP in extremely high regard, recounted how she attended her GP with a cough and was given medication which had not yet worked and she was still slightly worried about. Although the consultation sound only moderately enabling, she scored 11 out of a possible 12 on enablement.

### MYMOP

This section reports on the same cognitive processes for the 17 patients who completed MYMOP.

#### Comprehension process

Participants generally found MYMOP more difficult to complete than PEI. Despite an initial explanation that this should be for a single condition/problem, four people completed MYMOP for more than one condition. Eight respondents wrote the name of the condition instead of the symptom, although most of these could clearly define what symptom meant when asked. One respondent, who wrote “arthritis” as his symptom gave the following response when asked to explain what symptom meant to him:

*Patient 14: “Well symptom is, is well what’s happening with you, symptom is, is, is the problem you’ve got at the, at the moment. […], you know, you’ve got a rash. You know, that’s a symptom of something, it might be a stinging nettle, might be a drug you’ve taken, but to me that’s a symptom. […] So to me the symptom is the aches, the pains. […]”*

*INT: “Yeah, and is there a reason you would write arthritis rather than aches and pains?”*

*Patient 14: “Yeah because that’s what I’ve been told. That’s what it is.”*

Two respondents associated the word activity with sport and one initially felt that, as he was retired, he did not have any important activities, because nothing he did was essential. However, most understood activity as doing anything they enjoyed doing or had a responsibility to do. There was a very strong convergence of well-being as relating to the whole person, mind and body.

#### Recall process

The recall period for MYMOP is 1 week. Some respondents found difficulty in averaging a variable symptom over a week. One woman, with an allergic rash, hesitated and then circled response 2, explaining:

*Patient 8: “A week is quite a long time sometimes for little conditions like this … if you’d asked me the day that it erupted and the following day, I’d have said well it stops me doing it completely. But then … after that, it … no, it sort of gradually … you know, there were times in the day when no problem whatsoever. And then now, you know, it’s just spot there and a small spot there.”*

*INT: “So is what you’re saying, the two doesn’t really give a fair picture […] of something that […] has gone from six […] to zero?”*

*Patient 8: “To zero, yes.”*

#### Response process

MYMOP has a seven-point Likert scale from 0 to 6 labelled “as good as it could be” at one end and “as bad as it could be” at the other. Patients differed in their interpretation of the top endpoint, with some interpreting it as asymptomatic and others meaning as good as possible, given their knowledge of their own health condition. Other patients used the bottom of the scale as an anchor for as bad as their problem had ever been, and one used the middle of the scale as an anchor for his average symptom level.

#### Face validity

In general, despite initially finding it more difficult, most people seemed to find MYMOP more face valid than PEI. Nine respondents thought the questionnaire was relevant to them, as opposed to three who thought it was not and five who were neutral. Those who found it applicable liked that it measured status directly and did not require an assessment of change in status due to a doctor’s appointment. As one respondent put it “*This one was about the doctor (PEI). This one was about me (MYMOP)”* (Patient 16). Some participants appreciated that it was individualised and measured well-being, although some questioned whether well-being was likely to be changed by a single GP appointment.

## Discussion

### Key findings

The PEI questions were open to many different interpretations. The results are in line with previous research findings [47] that transitional instruments like the PEI are more difficult than status questionnaires for respondents to complete, because they require a greater number of internal calculations. The format of the PEI, which explicitly asks about the role of the doctor, may make it particularly prone to hypothesis-guessing: some patients seemed to give their response based on their own assessment of GP performance rather than their enablement. This is consistent with research that shows patients will give high satisfaction scores for negative experiences, if they perceive any failures were not the doctor’s fault [48].

Lastly, the merging of two response categories into one (same or less) on the PEI scale caused unnecessary confusion, which is an important lesson for future questionnaire development.

**Fig. 3:**
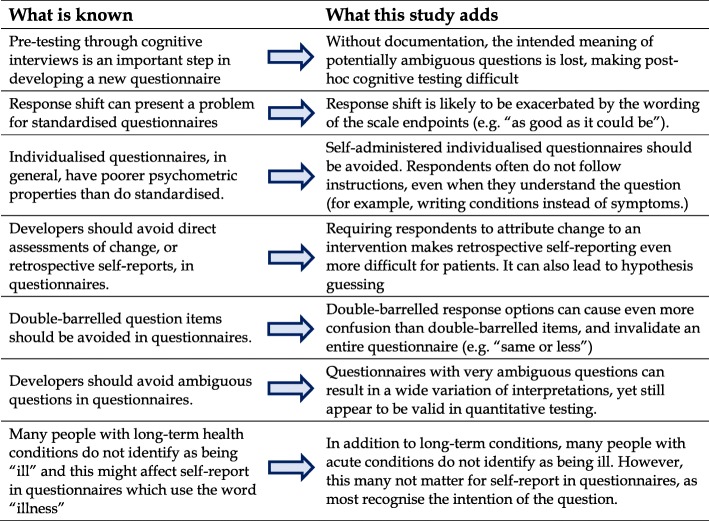
What this study adds

The individualised nature of MYMOP appealed to people completing it. However, the difficulty people had in adhering to the instructions of sticking to one condition and naming a symptom, not a condition, suggests that MYMOP, or similar instruments, would be very difficult to administer outside an interview. The scale of MYMOP, anchored with “as good as it can be” may lend itself to response shift, [49] as patients recalibrate their expectation of illness, particularly those with long-term conditions. Figure 3 shows a summary of what this research adds to what is already known on this subject.

### Strengths and limitations

The key strength of this study is that it has provided valuable findings which can inform users of these two PROMs and PROM developers more generally. These findings are based on an established model of cognitive theory [18, 19]. There were some weaknesses with this study. The majority of interviews were with female, white participants. Although the proportion of ethnic minorities was representative of the UK population outside London, it would have been preferable to have a greater ethnic variation in the sample. Most of the coding was carried out by a single researcher. The two co-researchers did independently review four transcripts to inform the coding framework and reviewed the final coded data against this framework; nonetheless, independent coding of all transcripts may have provided a more rigorous analysis.

The use of the same patients as the previous qualitative study is a weakness, because it is likely that the cognitive interviews were *reactive* [47] to the qualitative interviews carried out immediately prior: i.e. patients’ response to the two questionnaires could have been affected, as they had already reflected on the outcomes from the consultation.

Lastly, neither questionnaire was used exactly as recommended. PEI is designed to be completed straight after a consultation but, in this study, patients completed it 1 day to 3 weeks after their consultation. A key problem with such reterospective self-reporting is that respondents often do not remember their baseline health state; [47] many participants who do not accurately recall a prior health state will attempt to construct or guess a response [50]. MYMOP is designed to be completed through interview, but this study tested self-completion. However, PEI and MYMOP have both been adapted for use in this way, [39, 51] because self-completion straight after a consultation or completion through interview is not always possible in research.

### Comparison with literature

Some of the issues found have also been reflected in earlier studies. The problem of retrospective survey accuracy has been widely recognised [47] and this is being addressed by increasing use of digital methods to capture information about patient’s current health status in real-time [52, 53]. As in the current study, Paterson found that, when completing PEI sometime after an intervention, patients had difficulty attributing change to the intervention; that the PEI had lower face validity than MYMOP when used for chronic conditions and that such patients did not see their problems as an “illness” [54], a finding which has been observed in cognitive interviews of other related questionnaires [55, 56]. In a large study of GPAQ data, Mead et al. found 16% of PEI scores had at least one “not applicable” response [27]. Mead and Bower suggested this might be due to consultations in which enablement is not an explicit feature, such as those for repeat prescriptions, and that further research was required to potentially ascertain other reasons. The current study has found that the ambiguity of the double-barrelled “same or less” is at least partly contributory to respondent’s overuse of the “not applicable” category.

Despite some problems with face validity, the PEI has been widely translated [24] and validated [15, 24, 27–29]. Testing shows that enablement and satisfaction are related but distinct constructs [28]. In investigating this, Mercer et al. found that there could be empathy without enablement, but there was no enablement without empathy [57]. Although this is consistent with the importance of empathy in the therapeutic relationship, it is feasible to think of consultations where patients were not satisfied, yet were enabled. Mercer’s findings are also consistent with the finding in the current study, of hypothesis-guessing on the part of patients. Two patients in the current study described consultations which did not sound particularly enabling, yet had high enablement scores. Both patients had high regard for their GP. They justified these scores through verbal report and appeared to understand the questions. In contrast, three patients described consultations which did sound enabling, but had low enablement scores (1–2). One of these had low regard for her GP, and the other two felt that the questions (e.g. “cope with life”) were at odds with their reasons for seeking care. These complications may partly explain some non-intuitive results in quantitative testing of the PEI [58, 59].

As found in other studies [54] the format of MYMOP appealed to patients more than the PEI and carried greater face validity. MYMOP, and nearly all other individualised questionnaires, are recommended to be administered in interview [30, 33, 60] and this research has confirmed this recommendation. The scale of MYMOP, anchored with “as good as it can be” may lend itself to response shift, [49] as patients recalibrate their expectation of illness, particularly those with long-term conditions. Patients also experienced problems scoring minor conditions with rapidly changing symptoms, given the requirement to score how the condition has been over the last week. This also serves to highlight the issue of whether it is relevant to focus only on symptoms in something short-term or self-limiting, as any change measured is likely to be positive. The domains of understanding, reduction of concern and ability to help self in the future may be more important to patients with acute or minor illnesses than current symptoms (which may have resolved within a week). Furthermore, primary care patients frequently present with problems unrelated to symptoms or function, [4] and many primary care patients have multiple long-term conditions [5, 61, 62]. As their function may not improve, experts have suggested the need to measure wider outcomes in such patients, such as a sense of control and the ability to self-care [63].

## Conclusions

The current study was originally designed to inform development of a new PROM for primary care. The insights which informed the development of this PROM are transferable to both users and developers of individualised and transitional questionnaires.

Users should heed the developer’s recommendation that MYMOP should be administered through interview [64]. The scale of MYMOP may lend itself to response shift, which should be taken into account if using it to measure change over time from a point baseline. The PEI, although widely used and translated in different countries for primary care, lacks face validity for some patients with chronic conditions and may be open to hypothesis-guessing. When administered days or weeks after a consultation, it may be more difficult for patients to complete than the immediate post-consultation version.

For developers, this study has confirmed that cognitive interviews can uncover problems that cannot be uncovered through quantitative psychometric testing, even with existing questionnaires that are in widespread use. Many such questionnaires were developed without comprehensive cognitive testing and lack clear documentation about the intended meaning of questions, which makes post-hoc cognitive testing difficult. Cognitive interviews should be an essential part of new measure development, to ensure the questions are understood consistently and are measuring the desired concept.

## Additional file

Additional file 1: Verbal Probing Schedule for PEI and MYMOP. List of verbal probes used for the cognitive interviews with PEI and MYMOP respectively. (DOCX 23 kb)

## Abbreviations

EQ-5D: European Quality of Life-5 Dimensions
MYMOP: Measure Yourself Medical Outcomes Profile
PEI: Patient Enablement Instrument
PROM: Patient-reported outcome measure
SF-36: Short Form 36
