# Supplementary material for: Patient understanding of two commonly used patient reported outcome measures for primary care: a cognitive interview study
Source: BMC Fam Pract. 2018 Sep 27;19:162. doi: 10.1186/s12875-018-0850-2 (PMC6161379; doi:10.1186/s12875-018-0850-2)
Supplement: Supplementary file 1 — Verbal Probing Schedule for PEI and MYMOP. List of verbal probes used for the cognitive interviews with PEI and MYMOP respectively. (DOCX 23 kb) [file 12875_2018_850_MOESM1_ESM.docx]

**Verbal Probing Schedule for PEI and MYMOP**

Researcher explanation:

*I’m going to ask you to complete two questionnaires in turn. I want to talk to you as you do it about why you are giving the answers you give. You see, we want to test how people interpret the questions, because two different people can interpret the same question in completely different ways. What I want to find out is what each question means to you, and why you give the response that you give for each question. So I’ll ask you some questions after you complete each questionnaire about how you are interpreting it. Some people find it easier to just “think aloud” and as they complete the questionnaire talk though the thought processes going on, without me asking questions. That’s fine too if you want to do that. Remember, the purpose of this is to test the questionnaire – not to test you, so there are no wrong answers. Are you ready to start?*

**Patient Enablement Instrument**

For each question:

Initial probe: Why did you give that answer?

If answers are not self-evident from this, additionally use probes below:

| 1. **Able to cope with life** | |
| --- | --- |
| What did question mean to you? |  |
| What do you understand by “cope”? What might “not coping” look like? |  |
| Why did you put? Better / much Better / Same or less / NA? |  |
| 1. **Able to understand your illness** | |
| What did the question mean to you? |  |
| What do you understand by “illness”? |  |
| What does “understand” mean to you in this context? |  |
| Why did you put? Better / much Better / Same or less / NA? |  |
| 1. **Able to cope with your illness** | |
| What did question mean to you? |  |
| What does “cope” mean to you in this context? Same as before? What might “not coping” look like? |  |
| Why did you put? Better / much Better / Same or less / NA? |  |
| 1. **Able to keep yourself healthy** | |
| What did question mean to you? |  |
| What do you think it means by “healthy”? |  |
| What sort of things might the question be referring to that people do to keep themselves healthy? |  |
| Why did you put? Better / much Better / Same or less / NA |  |
| 1. **Confident about your health** | |
| What did question mean to you? |  |
| What do you understand by “confident”? What might it feel like to be unconfident? |  |
| Why did you put? Better / much Better / Same or less |  |
| 1. **Able to help yourself** |  |
| What did question mean to you? |  |
| What do you think it means by “helping yourself” – give examples. |  |
| Why did you put? Better / much Better / Same or less / NA |  |
| 1. **Overall** |  |
| Is it easy or hard to remember, given that it was 2 weeks ago? |  |
| Did you find it easy to understand the scale?  When might you put N/A as opposed to Same or less, or much better as opposed to better? |  |
| Did you find it easy to understand all the questions? I noticed you hesitated at QX.... |  |
| Are you happy with all the responses, or some you thought maybe you could have ticked a different box? |  |
| General Comments made not in response to probes |  |

**MYMOP**

To be completed based on their health today or, if this is not possible, for their health at the consultation they were recruited at.

Answers based on: ...............................................................................................

For each question:

Initial probe: Why did you give that answer?

If answers are not self-evident from this, additionally use probes below:

| 1. **SYMPTOMS** | |
| --- | --- |
| What does the word symptom mean to you? |  |
| Why did you choose those two symptoms? |  |
| Was it easy to think of symptoms? |  |
| Why have you given it a number X? |  |
| What do you think the top /bottom of the scale means? When would you put 0/6? |  |
| 1. **ACTIVITIES** |  |
| Why did you choose that activity? |  |
| If no activity chosen – “but you said earlier that problem stopped you from X .....?” |  |
| Why have you given it a number X? |  |
| 1. **WELL-BEING** |  |
| What do you think well-being means? |  |
| Why have you given it a number X? |  |
| 1. **Overall** |  |
| Did you find that easy or difficult to complete? |  |
| How did it compare to the first one in terms of a) easiness |  |
| b) relevance to you  (Do you think it represents how you have been this week?) |  |
| How did you make your response apply over a week given that it might change over that week? |  |
